# Synthesis of Fructooligosaccharides by IslA4, a truncated inulosucrase from *Leuconostoc citreum*

**DOI:** 10.1186/s12896-015-0116-1

**Published:** 2015-02-07

**Authors:** Arlen Peña-Cardeña, María Elena Rodríguez-Alegría, Clarita Olvera, Agustín López Munguía

**Affiliations:** Departamento de Ingeniería Celular y Biocatálisis, Instituto de Biotecnología, Universidad Nacional Autónoma de México, Cuernavaca, Morelos C.P. 62210 México

**Keywords:** Fructosyltransferase, Fructooligosaccharides, Inulin, Inulosucrase, *Leuconostoc citreum*

## Abstract

**Background:**

IslA4 is a truncated single domain protein derived from the inulosucrase IslA, which is a multidomain fructosyltransferase produced by *Leuconostoc citreum*. IslA4 can synthesize high molecular weight inulin from sucrose, with a residual sucrose hydrolytic activity. IslA4 has been reported to retain the product specificity of the multidomain enzyme.

**Results:**

Screening experiments to evaluate the influence of the reactions conditions, especially the sucrose and enzyme concentrations, on IslA4 product specificity revealed that high sucrose concentrations shifted the specificity of the reaction towards fructooligosaccharides (FOS) synthesis, which almost eliminated inulin synthesis and led to a considerable reduction in sucrose hydrolysis. Reactions with low IslA4 activity and a high sucrose activity allowed for high levels of FOS synthesis, where 70% sucrose was used for transfer reactions, with 65% corresponding to transfructosylation for the synthesis of FOS.

**Conclusions:**

Domain truncation together with the selection of the appropriate reaction conditions resulted in the synthesis of various FOS, which were produced as the main transferase products of inulosucrase (IslA4). These results therefore demonstrate that bacterial fructosyltransferase could be used for the synthesis of inulin-type FOS.

## Background

Fructooligosaccharides (β2-1, FOS) with a degree of polymerization (DP) in the range of 2–10, are one of the most important sources of carbon and energy for the beneficial microbiota (probiotics) that exist in the intestinal lumen of humans [1]. FOS are generally consumed directly as part of a healthy diet, including fruit and vegetables. FOS can also be consumed indirectly through functional foods, which can be enriched with FOS obtained by the hydrolysis of chicory inulin or produced industrially through enzymatic processes with commercial fructosyltransferases (FTFs) from fungal species like *Aspergillus* [2]. In the latter case, fungal enzymes are the preferred choice of enzyme because of their high specificity towards low molecular weight inulins, low hydrolytic activity and high stability [3,4]. In contrast, bacterial inulosucrases can be used to synthesize high molecular weight inulin with low specificity towards FOS production [5-7]. FTFs (EC 2.4.1.-) are enzymes that catalyze the transfer of the fructosyl moiety of sucrose to different acceptors, resulting in the synthesis of fructans with different molecular weights depending on the specificity of the enzyme. The fructosyl unit can also be transferred to water, resulting in the hydrolysis of sucrose. According to the classification system for carbohydrate-active enzymes, bacterial FTFs belong to family 68 of the glycoside hydrolases (GH68). Most FTFs are 45 to 64 kDa in length and consist of a single catalytic domain with a five-bladed β-propeller fold that encloses a funnel-like central cavity where the conserved catalytic residues are located. FTFs can be classified as inulosucrases (EC 2.4.1.9), which synthesize β2-1 linked fructans (inulin), or levansucrases (EC 2.4.1.10), which produce fructans with β2-6 linkages (levan) [8].

We previously reported the isolation of inulosucrase from *Leuconostoc citreum* CW28. This particular enzyme was designated IslA and was found to synthesize high-molecular-weight inulin. IslA is a multidomain enzyme containing additional regions at both the amino- and carboxyl-terminals of its catalytic domain, which are similar to those found in glucosyltransferases [9]. In this context, IslA4 is a truncated form of IslA that retains only the five-bladed β-propeller catalytic domain. IslA4 is therefore similar to several other fructosyltransferases previously reported in the literature, including the levansucrases from *Bacillus subtilis* (SacB) and *Gluconacetobacter diazotrophicus*, and the InuJ inulosucrase from *Lactobacillus johnsonii* NCC 533*.* We previously studied the effects of the additional domains of IslA on its overall properties and found that IslA4 was similar to IslA, in the sense that it produced mainly high molecular weight inulin. However, IslA4 exhibits a much higher hydrolytic activity than IslA under the same reaction conditions [10]. Interestingly, IslA developed a high level of hydrolytic activity following the elimination of some of its additional domains, and achieved similar activity to a single domain fructosyltransferase, such as levansucrase SacB, which can hydrolyze as much as 80% of the sucrose substrate [11]. It has been demonstrated that reaction specificity (i.e., hydrolysis or transferase) as well as product specificity (i.e., type and size of fructan or FOS) in fructosyltransferases is strongly dependent on the reaction conditions, including the substrate concentration and temperature [12,13], the form in which the enzyme is applied, such as free or immobilized [14], the presence of organic solvents or co-solvents [15], and the source of the enzyme [16].

In this study, we have evaluated the effect of the reaction conditions on the specificity of IslA4 and the truncated form of inulosucrase IslA, in an attempt to identify efficient enzymes for the synthesis of inulin-type FOS.

## Results and discussion

### Influence of substrate and enzyme concentration on IslA4 reaction specificity

**Figure 1:**
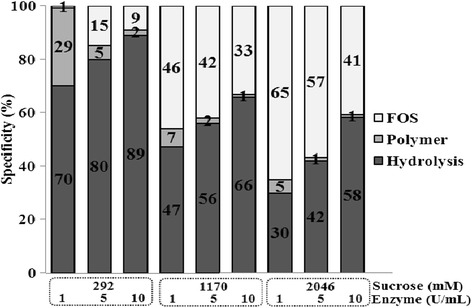
**Transferase efficiency of IslA4 as a function of enzyme and substrate concentration.** All reactions are carried out at 30°C in 50 mM phosphate (pH 6.0) containing 1 mM CaCl_2_ and allowed to proceed until they reached a sucrose conversion of approximately 90%.

A common characteristic of fructosyltransferases is their ability to transfer the fructosyl moieties of a substrate to an acceptor molecule (the fructan growing chain) or water, leading to the hydrolysis of the substrate. The effects of the IslaA4 and sucrose concentrations on the transfer and hydrolysis reactions of the fructosyl moiety are shown in Figure 1. As in many other FTF cases, transferase activity is favored by high substrate concentrations, because of the higher amount of sucrose molecules available with respect to water for the initial transfer of the fructosyl residue [17]. Similar behavior has also been reported for levansucrase from *B. subtilis*, where almost 80% of the sucrose was used for transferase reactions at sucrose concentrations greater than 1,750 mM [18]. In contrast, SacB becomes predominantly hydrolytic in terms of its activity at low sucrose concentrations (12 mM) [19]. Furthermore, levansucrase from *G. diazotrophicus* displayed only hydrolytic activity at sucrose concentrations lower than 60 mM [20]. An inverse reaction specificity effect was observed for the enzyme concentration of IslA4, where an increase in the enzyme concentration from 1 to 10 U mL^−1^ led to an increase in the level of hydrolytic activity, regardless of the sucrose concentration. Reactions involving a low enzyme concentration and high substrate concentration (e.g., 1 U mL^−1^ and 2,046 M, respectively) were therefore determined to be suitable reaction conditions for high transferase efficiencies, despite the lengthy reaction times required. In contrast, a high IslA4 concentration (10 U mL^−1^) coupled with a low sucrose concentration (292 mM) led to 90% of the sucrose being hydrolyzed. Similar behavior has also been reported for several other enzymes, such as SacB from *B. subtilis*, which transferred 78% of 1,750 mM sucrose at low enzyme concentrations [18].

With regard to other inulosucrases, it has been reported that the transfructosylation activity of *L. reuteri* 121 increased when the substrate and enzyme concentration were increased from 200 to 1800 mM and 40 to 130 U mg^−1^, respectively, with FOS being identified as the main transferase product together with a relatively small amount of polymeric inulin material. In contrast, the same enzyme becomes predominantly hydrolytic in its activity at concentrations lower than 200 mM [21]. Similar results have also been reported for IslA, which became increasingly efficient in terms of ability to synthesize inulin when the initial concentration of sucrose in the reaction medium was increased [13]. Several other partially truncated forms of IslA have also exhibited similar reaction specificity behavior [10].

An extreme example of this behavior is levansucrase SacB, which shares 39% identity with inulosucrase IslA4. In this case, the enzyme concentration has a much stronger influence on the reaction specificity, with the hydrolysis of 300 mM sucrose increasing from 67 to 98% at 35°C when the enzyme activity is increased from 1 to 5 U mL^−1^ [18].

### Product profile analysis

#### TLC analysis

**Figure 2:**
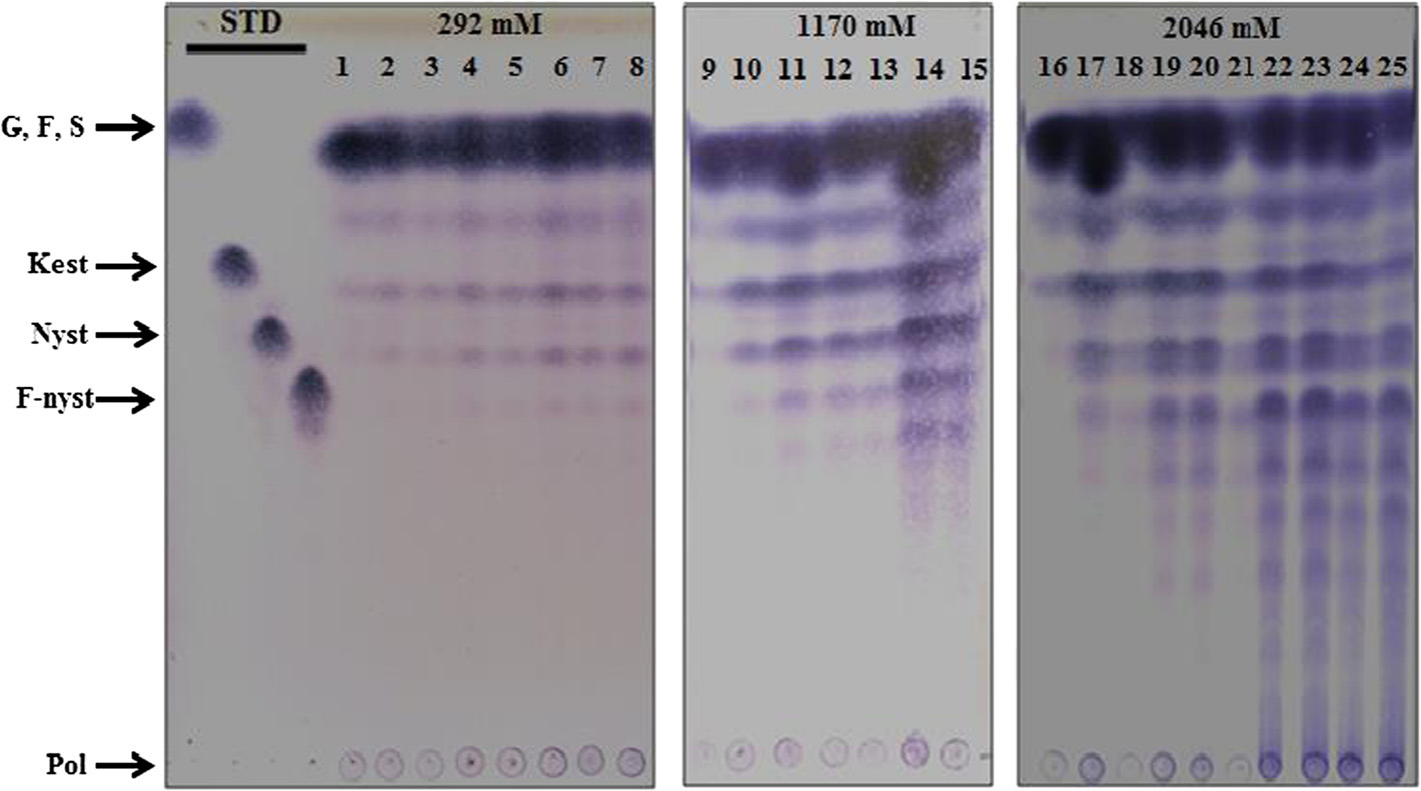
**Influence of substrate concentration on the IslA4 product profile, as observed by TLC.** All of the reactions are carried out with 1 U mL^−1^ of IslA4 at 30°C in 50 mM phosphate (pH 6.0) containing 1 mM CaCl_2_, and were allowed to proceeded to a sucrose conversion of approximately 90%. Samples were taken at different time points. The numbers in the figures correspond to the following reaction times: *292 mM* (0, 2, 4, 6, 8, 10, 12 and 24 h); *1170 mM* (0, 3, 6, 9, 12, 25 and 34 h); and *2046 mM* (0, 3, 6, 9, 12, 24, 27, 30, 34 and 48 h). The STD lane contained the following standards: G, glucose; F, fructose; S, sucrose; Kest, 1-kestose (GF2); Nyst, 1,1-nystose (GF3); F-nyst, fructofuranosyl-nystose; and Pol, polymer.

The most interesting aspect of the product specificity exhibited by IslA4 is that although IslA is responsible for the synthesis of high molecular weight inulin, the combination of a truncated domain with the appropriate reaction conditions can results in the synthesis of a wide variety of FOS as the major transferase products. This effect can be readily visualized by TLC analysis, as shown in Figure 2. In this particular case, the FOS and inulin could both be clearly seen amongst the final reaction products when the products of reactions containing 1 U mL^−1^ of enzyme activity are analyzed at three different initial sucrose concentrations. A direct comparison with standard compounds suggested that 1-kestose (β-D-fructofuranosyl-(2 → 1)-β-D-fructofuranosyl-(2 → 1)-α-D-glucopyranoside) would be the first acceptor product, and that this product form as a consequence of a fructosyl transfer reaction to sucrose, followed by 1-nystose (β-D-fructofuranosyl-(2 → 1)-β-D-fructofuranosyl-(2 → 1)-β-D-fructofuranosyl-α-D-glucopyranoside) and f-nystose (β-D-fructofuranosyl-(2 → 1)-β-D-fructofuranosyl-(2→1)-β-D-fructofuranosyl-(2→1)-β-D-fructofuranosyl-α-D-glucopyranoside), which were only observed after an extended reaction time. However, an increase in the substrate concentration led to an increase in FOS synthesis, which was accompanied by an increase in transferase activity. Furthermore, there was a shift in the product profile with FOS with a low DP being observed at a sucrose concentration of 292 mM (mainly 1-kestose and nystose), while FOS with a DP greater than 10 fructose units were obtained at a sucrose concentration of 2,046 mM. Inulin was formed as the major synthetic product under the standard reaction conditions (i.e., 292 mM sucrose and 1 U mL^−1^ of enzyme) using both IslA and its truncated form IslA4. Furthermore, the various FOS already described above were only observed as a small part of the product stream [10,22]. The reaction conditions can therefore be defined in detail to considerably reduce the inulin concentration in favor of forming FOS. In the case of *L. reuteri* 121 inulosucrase, 1-kestose and nystose were reported to be the only products obtained from 263 mM sucrose after 17 h of reaction [5]. However, an increase in the sucrose concentration to 840 mM led to the formation of additional FOS with a DP greater than five, regardless of the inulin concentration [23]. In the same context, InuJ and InuGA, which are inulosucrases from *L. johnsonii* NCC 533 [6] and *Lactobacillus gasseri* DSM 20143 [7], respectively, can synthesize inulin together with a wide range of FOS with DP values greater than five after long reaction times in reactions containing 600 mM of sucrose. Similar behavior has also been reported for *Zymomonas mobilis* levansucrase, where the product specificity shifted from levan to FOS when the sucrose concentration was increased to 800 mM [23]. Levansucrase from *Lactobacillus sanfranciscensis* TMW and a truncated version of the same protein also exhibited similar behavior [24].

One important thing to consider regarding the IslA4 reaction mechanism is that the low molecular weight FOS products appear to be synthesized by a non-processive mechanism. In this case, all of the FOS accumulating in the solution were derived from 1-kestose, which is the first reaction product formed when sucrose is used as an acceptor (Figure 2). It has been proposed previously that subsites +2 and +3, further away from the cleavage site (−1 and +1), could have a low affinity for the growing FOS chains, and that these sites would then release the product once the fructosyl unit had been transferred, representing the basis of a non-processive mechanism [21]. However, this mechanism does not appear to be applicable to the case of inulin, which is most likely synthesized through a processive mechanism because inulin is only observed in the reaction medium in this case as a high molecular weight product.

#### GPC and HPAEC-PAD analysis

**Figure 3:**
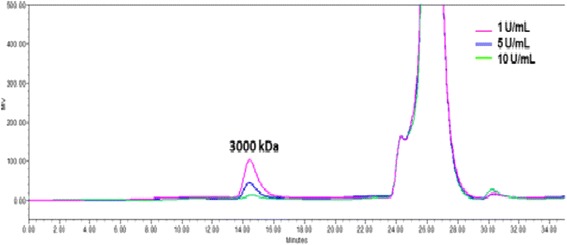
**Influence of enzyme concentration on the molecular weight distribution of inulin synthesized by IslA4 as observed by GPC.** Reactions were carried out at three different enzyme concentrations (i.e., 1, 5 and 10 U mL^−1^ of IslA4) with 292 mM sucrose at 30°C in 50 mM phosphate (pH 6.0) containing 1 mM CaCl_2_, and were allowed to proceed until they reached a sucrose conversion of approximately 90%.

The effect of the enzyme concentration and the substrate to enzyme ratio on the molecular weight distribution of the inulin synthesized by IslA4 was analyzed by GPC, and the results are shown in Figure 3. This result clearly shows that a product with an average molecular weight of 3,000 kDa was obtained following 14 min of elution. The distribution and size of the inulin produced was therefore independent of the enzyme concentration and of lower molecular weight than inulin produced by *L. reuteri* 121 [5] and *L. johnsonii* NCC 533 [6], which reached molecular weights as high as 10,000 and 40,000 kDa, respectively. This result also revealed that high enzyme concentrations favored the hydrolysis reaction, which resulted in the synthesis of small amounts of inulin (Figure 1). This behavior can therefore be explained in terms of the interactions between the enzyme molecules or enzyme-polymer molecules rather than the availability of active sites for a given sucrose concentration.

**Figure 4:**
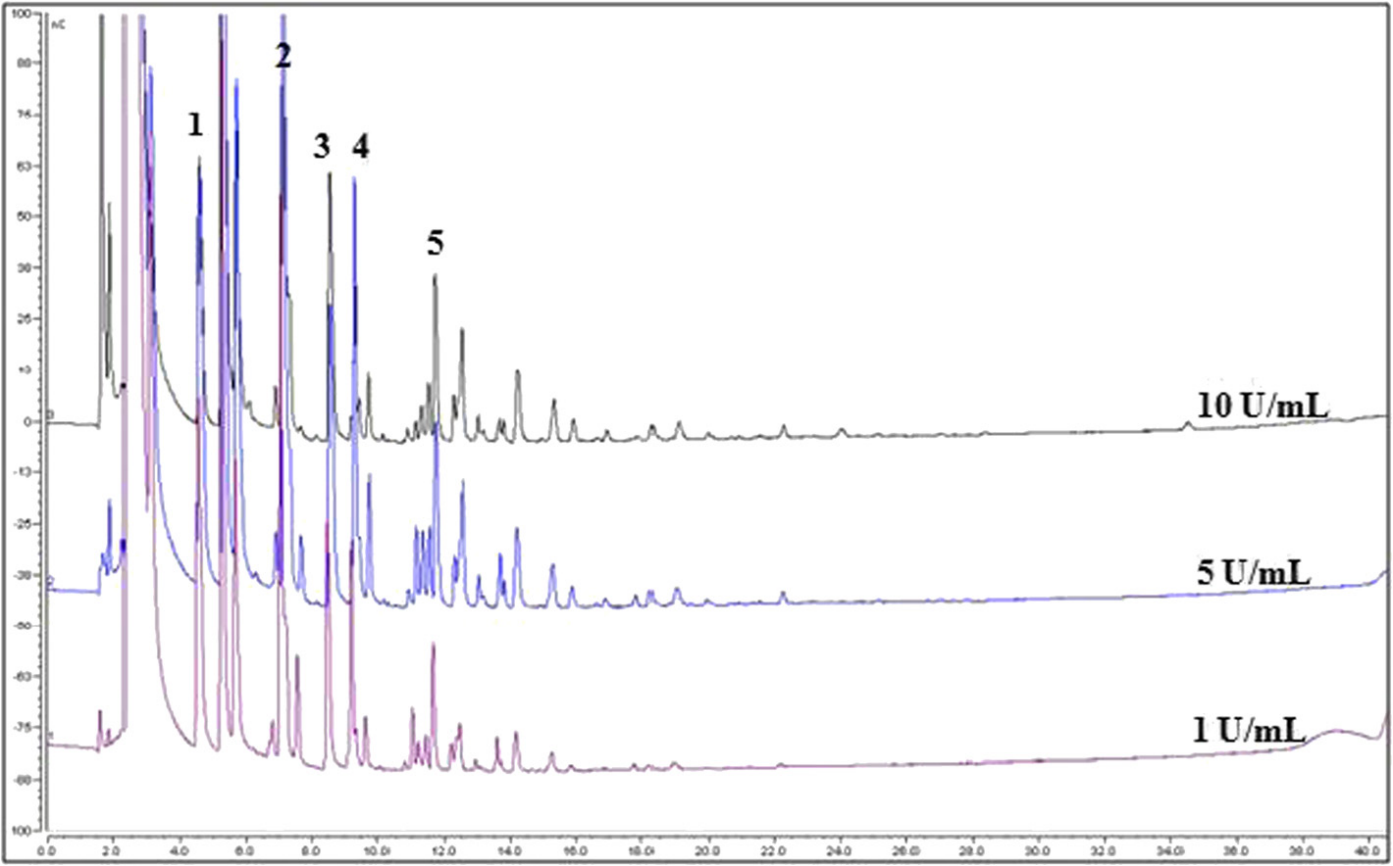
**Influence of enzyme concentration on products synthesized by ISlA4 as observed by HPAEC-PAD analysis.** Reactions were carried out at three different enzyme concentrations (i.e., 1, 5 and 10 U mL^−1^ of IslA4) with 292 mM of sucrose at 30°C in 50 mM phosphate (pH 6.0) containing 1 mM CaCl_2_, and were allowed to proceed until they reached a sucrose conversion of approximately 90%. Identified oligosaccharides: 1) 1-kestose; 2) 6-kestose; 3) neokestose; 4) nystose; and 5) f-nystose.

HPAEC-PAD analysis allowed for the identification of the synthesized FOS. An HPAEC-PAD chromatogram of the products obtained at three different enzyme concentrations is shown in Figure 4. Based on the retention times of the standards, the signals obtained at 4.5, 7.0, 8.5, 9.0 and 11.5 min were attributed to 1-kestose, 6-kestose (β-D-fructofuranosyl-(2→6)-β-D-fructofuranosyl-(2→6)-α-D-glucopyranoside), neokestose (β-D-Fructofuranosyl-(1→6)-β-D-fructofuranosyl-α-D-glucopyranoside), nystose and f-nystose, respectively. Although the amperometric detection intensity is not always proportional to the amount of product, the amount of synthesized FOS decreased in the current system as the molecular weight of the product increased, in a similar manner to that reported for Inu from *L reuteri* 121 [5]. This effect can be observed quantitatively in Figure 2. Unidentified products were eluted between 1-kestose and 6-kestose (products 1 and 2), and between nystose and f-nystose (products 4 and 5). Although these products have not yet been identified, the results suggest that the smallest FOS obtained after an elution time of 5 min wa*s blastose*

(β-D-fructofuranosyl-(6 → 2)-α,β-D-glucopyranoside), which is an isomer of sucrose. Blastose could be produced by the direct transfer of fructose to glucose in C6 or by the hydrolysis of the trisaccharide neokestose (product 3), which is a transfer product resulting from the transfer of fructose to the C6-position of the glucose unit in sucrose. Interestingly, these unidentified products were also reported in inulosucrase from *L. reuteri*121 [5] and *L. johnsonii* NCC 533 [6]. However, the formation of these products does not represent a general property of inulosucrases, because these products were not observed in InuGA and InuGB from *L. gasseri*, where linear β 2-1 FOS was the only FOS obtained from the direct transfer of fructose to the C1-position of the fructose acceptor [7].

**Figure 5:**
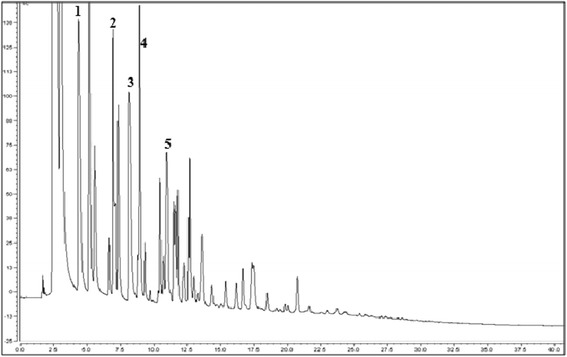
**IslA4 product profile under high transferase conditions as observed by HPAEC-PAD.** Reactions were carried out with 2,046 mM of sucrose and 1 U mL^−1^ of IslA4. The known oligosaccharides have been indicated as follows: 1) 1-kestose; 2) 6-kestose; 3) neokestose; 4) nystose; and 5) f-nystose.

The highest transferase yield for the reaction was obtained at the highest sucrose concentration and the resulting FOS profile is shown in Figure 5. Once again, the reaction led to the formation of large amounts of 1-kestose, 6-kestose, neokestose, nystose and f-nystose, as well as a large amount of FOS with a DP >5. Despite the limitations of the separation equipment, several products were observed with retention time in excess of 20 min. This behavior was similar to that reported for *L. reuteri* 121, which gave FOS with a higher DP than nystose when it was synthesized under high substrate concentrations [21].

### Quantitative analysis of the inulin and FOS synthesized by IslA4

The amount of fructose transferred and incorporated into inulin (F_POL_) was determined from a reference plot relating inulin GPC elution area to inulin concentration, which was constructed according to the procedure described above using inulin precipitated with ethanol and subsequently freeze-dried. With this methodology in hand, it was possible to demonstrate that although the inulin synthesis was independent of the substrate concentration, this factor is still an important function of enzyme concentration and activity. Overall, the level of inulin synthesis was found to be negligible at high enzyme concentrations, as shown in Table 1. These results therefore suggested that the increase in transferase activity that results from the rise in substrate concentration could be attributed to the exclusive synthesis of FOS, as already shown by TLC and HPAEC-PAD (Figures 2 and 4).

**Table 1:** **Quantitative analysis of the products synthesized by IslA4 under different reaction conditions used**

**Sucrose (mM)**	**IslA4 (U mL** ^**−1**^ **)**	**Sucrose consumed (%)**	**F**_**POL**_ **(mM)**	**F**_**FOS**_ **(mM)**
	1	92 ± 1.11	95 ± 4.3	3 ± 6.78
292	5	90 ± 1.37	18 ± 0.4	49 ± 2.56
	10	93 ± 0.96	6 ± 0.1	29 ± 1.50
	1	94 ± 0.45	87 ± 0.5	584 ± 0.12
1170	5	93 ± 1.59	27 ± 0.1	465 ± 0.21
	10	97 ± 1.67	11 ± 0.1	407 ± 0.10
	1	87 ± 3.40	89 ± 0.2	1251 ± 1.20
2046	5	90 ± 0.87	23 ± 0.2	1093 ± 0.18
	10	94 ± 2.81	5 ± 0.3	808 ± 0.05

Values shown are means ± SD of the results from three independent experiments.

In quantitative terms, it was possible to reduce the amount of inulin from 16 to 4 or 1 g L^−1^ by increasing the IslA4 concentration from 1 to 5 or 10 U mL^−1^ respectively, at any of the three sucrose concentrations studied. The highest amount of inulin produced under these reaction conditions was significantly higher than that reported for inulosucrase from *L. reuteri* 121, where only 0.8 g L^−1^ inulin was obtained from 90 g L^−1^ of sucrose. Although no explanation was offered for the result obtained in this particular case, the incubation times reported would only have allowed for an 18% conversion [5]. In the same context, the highest polymer yield (16 g L^−1^) obtained with IslA4 was very similar to the yield of inulin calculated for the inulosucrases InuGA and InuGB from *L. gasseri*, which produced 11 and 13 g L^−1^ of inulin, respectively, from their reactions with 205 g L^−1^ of sucrose [7]. In contrast, InuJ from *L. johnsonii* NCC 533 allowed for the synthesis of 1.5 g L^−1^ of inulin from 200 g L^−1^ of sucrose [6]. Taken together, these results suggest that IslA4 is a much more suitable enzyme for the synthesis of inulin than any of the other inulosucrases currently available, even at low substrate concentrations (292 mM), where hydrolysis is the dominant reaction pathway. However, based on the FOS concentration, F_FOS_ (mM), which can be indirectly calculated according to the method described above, a low concentration of F_FOS_ (mM) was obtained when the reactions were conducted at 292 mM, which was the lowest sucrose concentration evaluated (Table 1). As shown in Figure 1, these reaction conditions also induced high levels of hydrolysis, as exemplified by the reaction conducted with 10 U mL^−1^ of enzyme activity, where up to 89% of the substrate was converted to glucose and fructose. Furthermore, increasing the sucrose concentration from 1,170 to 2,046 mM in reactions with 1 U mL^−1^ of enzyme activity, led to a two-fold increase in the amount of fructose incorporated in the FOS, despite the presence of a large amount of hydrolyzed sucrose (around 600 mM) in the mixture.

Figure 1 provides a detailed overview of the fate of each transferred fructose, in terms of whether it goes to water, inulin or FOS. Based on this analysis, it can be concluded that the majority of the FOS synthesis was conducted from 2046 mM sucrose in reactions with 1 U mL^−1^ of enzyme, where 70% sucrose was used for transfer reactions, and 65% of this sucrose was directed towards a transfructosylation pathway for the synthesis of FOS. In contrast, the use of a low sucrose concentration (292 mM) led not only to a decrease in the transferase reaction but also favored polymer synthesis.

## Conclusions

An efficient set of reaction conditions have been established for the synthesis of FOS and/or inulin using IslA4 inulosucrase. In particular, the results of the current study have shown that the enzyme specificity can be shifted almost exclusively from the synthesis of high molecular weight inulin to the synthesis of FOS. This shift was made possible in this truncated form of the multidomain inulosucrase IslA through the careful selection of the enzyme and substrate concentration conditions. The results of the current study have also shown that the use of a high sucrose concentration in conjunction with a low IslA4 concentration results in the highly efficient synthesis of low molecular weight FOS, including 1-kestose, 6-kestose, neokestose, nystose and f-nystose.

## Methods

### Preparation of *Escherichia coli* cell extracts and purification of IslA4

**Figure 6:**
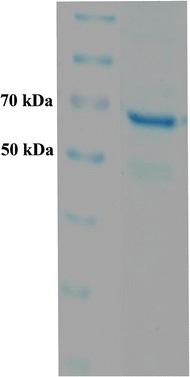
**SDS-PAGE of the purified IslA4 inulosucrase form.** The intracellular protein extracted from *E.coli*, was purified by affinity chromatography in Ni-nitroacetic acid (Ni-NTA) resin (Qiagen) and eluted with increasing amounts of imidazole. Afterwards, the enzyme was dialyzed against phosphate buffer and concentrated by ultrafiltration on an Amicon Ultra centrifugal Filter. 10,000 NMWL (Merck Millipore, Billerica, MA, USA).

*Escherichia coli* BL21 cells transformed with the pET28a-*islA4* gene were grown in Luria-Bertani broth containing 50 μg mL^−1^ kanamycin at 37°C and 120 rpm. The culture was induced with 0.2 mM isopropyl β-D-1-thyogalactopyranoside (Gold Biotechnology, Inc., St Louis, MO USA). Once the cells reached an optical density of 0.6 (600 nm) they were incubated at 18°C for 6 additional hours. The cells were then harvested by centrifugation (2,500 × *g* for 10 min) and the resulting pellet washed twice with a 50 mM phosphate buffer (pH 6.0) containing 1 mM CaCl_2_ before being re-suspended in 5 mL of the same buffer containing pepstatin (0.7 μg mL^−1^) and Complete (one tablet to 50 mL of cell extract), which were added as protease inhibitors, and sonicated. Cell debris was removed by centrifugation (21,130 × *g* for 30 min) and the supernatant containing the enzyme was recovered for further processing. The protein concentration was determined according to the Bradford method [25] using the Bio-Rad reagent, with bovine serum albumin (BSA) (albumin fraction V; Sigma Aldrich, St. Louis, MO, USA) being used as a standard. The enzyme was purified by His-tag affinity chromatography. A bed volume of 600 μL of Ni-nitroacetic acid (Ni-NTA) resin (Qiagen) was used to bind the protein from 5 mL of cell extract. The resin was equilibrated with 500 μL of pH 7.0 binding buffer containing 50 mM NaH_2_PO_4_, 300 mM NaCl and 10 mM imidazole. The cell extract was diluted with an equivalent amount of the binding buffer and incubated overnight at 4°C under agitation with the equilibrated resin. The proteins from the extract were subsequently eluted from the resin by successive additions of buffer, with increasing amounts of imidazole (i.e., 30, 60 and 100 mM of imidazole). Finally, the enzyme was eluted with buffer containing 250 mM imidazole. The protein was dialyzed against 50 mM phosphate buffer (pH 6.0) containing 1 mM CaCl_2_ and concentrated by ultrafiltration on an Amicon Ultra centrifugal Filter 10,000 NMWL (Merck Millipore, Billerica, MA, USA). The purity of IslA4 was verified by 10% SDS-PAGE (Figure 6); it shows an adequate level of enzyme purity.

### Activity assay

In order to have a general idea of the enzyme activity, initial reaction rates were measured at 30°C in 50 mM phosphate buffer (pH 6.0) containing 1 mM CaCl_2_ in the presence of 292 mM sucrose. The global activity was expressed as the reducing sugars released from sucrose using the 3,5-dinitrosalicylic acid method (DNS) [26]. One *global* activity unit (U) was defined as the amount of enzyme required to produce 1 μmol of reducing sugars per minute. The specific hydrolysis and transferase activities were measured by HPLC based on the amounts of fructose and glucose. The HPLC method used in the current study is described below.

### Influence of reaction conditions on IslA4 product and reaction specificity

The enzymatic synthesis was carried out at 30°C in 50 mM phosphate (pH 6.0) containing 1 mM CaCl_2_ at three different sucrose (292, 1,170 and 2,046 mM) and enzyme (1, 5 and 10 U mL^−1^) concentrations to evaluate the influence of the reaction conditions on the product and reaction specificities of IslA4. All of the reactions were allowed to proceed until they reached a sucrose conversion of approximately 90%. The reaction specificity (i.e., hydrolysis to transferase ratio) was determined by measuring the amounts of glucose and fructose released during the reaction. Free glucose (Gf) was derived from hydrolyzed sucrose as well as the sucrose that was used as a fructose donor for the transferring reactions, whereas free fructose (Ff) was derived exclusively from the hydrolytic activity of the enzyme. The difference between the amounts of consumed and hydrolyzed sucrose could therefore be used to determine the amount of fructose used for transfructosylation (Ft), i.e., the transferase activity.

### FOS, sugars and fructan analysis

#### TLC analysis of the oligosaccharide profile

The oligosaccharides produced by IslA4 were analyzed by TLC using silica gel 60 glass plates (Merck, Darmstadt, Germany). Samples were taken at different reaction times and spotted (1 μL) onto a TLC plate. Fructose, glucose and sucrose (Sigma Aldrich), and 1-kestose (GF2) nystose (GF3) and fructofuranosyl-nystose (Wako Pure Chemical Industries) were used as standards. The TLC plates were ran twice using a 15:9:6 (v/v/v) mixture of ethanol, butanol and water as the mobile phase. The plates were then air-dried and sprayed with an alcoholic solution of α-naphthol before being heated at 150°C.

#### Sugars analysis

Sugars were quantified by high-pressure liquid chromatography (HPLC) using a Waters 600E system controller (Waters Corp. Milford, MA, USA) equipped with a refraction index detector (Waters 410) using a Prevail Carbohydrate ES column (250 × 4.6 mm) at 30°C. The HPLC system was eluted with a mobile phase consisting of a 75:25 (v/v) mixture of acetonitrile and water at a flow rate of 1.0 mL min^−1^. The molecular weight of the Inulin was determined by gel permeation chromatography (GPC) using a serial set of Ultrahydrogel (UG 500 and linear) columns at 30°C. The columns were eluted with a mobile phase sodium nitrate 100 mM at a flow rate of 0.8 mL min^−1^. Oligosaccharides were separated and analyzed by high-performance anion exchange chromatography with pulsed amperometric detection (HPAEC-PAD, Dionex), using a CarboPac PAD-200 column (250 × 2 mm). The following gradient was used: eluent A at 100% (0 min), 99% (0.5 min), 80% (25 min), 20% (85 min) and 100% (95 min). Eluent A was 150 mM sodium hydroxide and eluent B was 150 mM sodium hydroxide in 500 mM sodium acetate.

### Quantitative analysis of the products synthesized by IslA4

The polymer was precipitated from the supernatant by the addition of 2 volumes of cold 96% ethanol followed by centrifugation (2500 × *g* at 30 min). The material was then dissolved in MilliQ water and dialyzed against 50 mM phosphate buffer (pH 6.0) containing 1 mM CaCl_2_, and the resulting polymers were freeze-dried and occupied to produce a standard curve, which was used to calculate the amount of fructose incorporated into the polymer (F_POL_). For quantitative purposes, the level of fructose incorporated exclusively into FOS (F_FOS_) was defined as the difference between Ft and F_POL_ (i.e., F_FOS_ = Ft − F_POL_). Although this method meant that the amount of fructose incorporated into FOS was calculated in an indirect manner, the results still allowed for a comparative analysis.

## Abbreviations

DP: Degree of polymerization
FTF: Fructosyltransferase
FOS: Fructooligosaccharide
GPC: Gel permeation chromatography
HPAEC-PAD: High performance anion exchange chromatography with pulsed amperometric detection
TLC: Thin-layer chromatography
